# Novel Foraging Behaviors of an Urban Bird, the Light‐Vented Bulbul (*Pycnonotus sinensis*)

**DOI:** 10.1002/ece3.72928

**Published:** 2026-01-07

**Authors:** Yuxin Li, Sidan Lin, Wei Liang

**Affiliations:** ^1^ Ministry of Education Key Laboratory for Ecology of Tropical Islands, Key Laboratory of Tropical Animal and Plant Ecology of Hainan Province, College of Life Sciences Hainan Normal University Haikou China

**Keywords:** artificial light at night, behavioral plasticity, novel behavior, *Pycnonotus sinensis*, urban birds, urbanization

## Abstract

Urban environments expose animals to the situation with a greater diversity of novel stimuli, often promoting the emergence of innovative behaviors. Such behavioral plasticity is considered a key strategy that aids birds in adjusting to urbanization. Identifying cases of behavioral innovation across different species and behavioral contexts is crucial for understanding the ways in which birds adapt to urban environments. This study reports two novel foraging behaviors observed in the Light‐vented Bulbul ( *Pycnonotus sinensis* ), a common urban bird in southern China. In the first case, Light‐vented Bulbuls were observed foraging on tree trunks in a woodpecker‐like manner to capture insects; in the second case, Light‐vented Bulbuls foraged on insect aggregations under artificial light at night. However, these behaviors have currently been observed only in a small number of individuals. It remains unclear whether these behaviors will become established through natural selection or spread throughout the population via social transmission and cultural inheritance, or they will extinguish with the individuals. Nevertheless, our findings provide new insights into possible behavioral adaptation strategies of Light‐vented Bulbuls to urban environments.

## Graphical abstract

Light‐vented Bulbul ( *Pycnonotus sinensis* ) were observed foraging on tree trunks in a woodpecker‐like manner. Light‐vented Bulbuls foraged on insect aggregations under artificial street light. Our findings provide new insights into possible behavioral adaptation strategies of Light‐vented Bulbuls to urban environments.

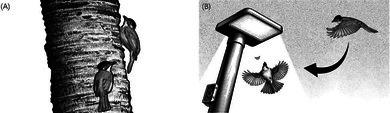

## 1 Introduction

With the expansion of urban landscapes and increasing anthropogenic disturbance, global avian species diversity (Newbold et al. 2015), phylogenetic diversity (Sol et al. 2017), and functional diversity (Sol et al. 2020) are undergoing rapid decline. However, different bird species do not respond to urbanization randomly. The filtering effect of urbanization preferentially removes “urban avoiders” that lack pre‐adaptations to urban environments, while retaining or promoting “urban utilizers” and “urban exploiters” that possess urban tolerance (Callaghan et al. 2019; Patankar et al. 2021; Hahs et al. 2023). Numerous studies have demonstrated that this selective filtering mechanism is mediated by avian traits, such as body size, clutch size, diet, and nest location (Møller 2009; Neate‐Clegg et al. 2023; Zhong et al. 2023; Lin and Liang 2025a). Among these traits, behavioral plasticity has emerged as a key factor influencing birds' ability to adapt to urban environments. For example, species with higher behavioral plasticity are more likely to exploit novel food resources (i.e., consumer innovations) or devise novel searching and foraging techniques (i.e., technical innovations) in urban settings (Ducatez et al. 2020). However, the macro‐ecological generality of behavioral plasticity has only recently garnered attention (Ducatez et al. 2020).

Behavioral plasticity determines whether birds can generate behavioral responses to cope with new or unusual challenges, such as food shortages and extreme climatic events (Sol 2003). Thus, behavioral plasticity can contribute to the maintenance of population fitness during environmental changes, consistent with the cognitive buffer hypothesis (Fristoe et al. 2017; Sayol et al. 2020). The novel behaviors observed in animals can serve as a metric to gauge the extent of their behavioral plasticity (Ducatez et al. 2020). Accumulating empirical evidence indicates that some birds can modify their behaviors to respond to urbanization challenges and opportunities. For example, Great Tits ( *Parus major* ) and Blue Tits ( *Cyanistes caeruleus* ) learned to peck through foil caps of milk bottles to consume the cream layer (Fisher and Hinde 1949; Hinde and Fisher 1951). Sulfur‐crested Cockatoos ( *Cacatua galerita* ) developed the ability to open garbage bins to access food (Klump et al. 2021, 2022) and to manipulate twist‐handle public drinking fountains for water (Klump et al. 2025). Therefore, identifying additional instances of behavioral innovation across different species and contexts is crucial for understanding how birds adapt to urban environments.

The Light‐vented Bulbul ( *Pycnonotus sinensis* ) is widely distributed across southern China (Zheng 2023) and is a common urban bird species (Lin and Liang 2025b). It is a diurnal bird species that often forms small flocks, perching conspicuously atop shrubs and trees (Zhao 1999; Fishpool and Tobias 2020). The Light‐vented Bulbul is an omnivorous bird, predominantly consuming berries and fruits from plants. In winter, among the northern populations of the Light‐vented Bulbul, plants constitute 96.67% of their total diet, while insects account for a mere 0.95% (Guo 2018). In contrast, the southern populations have a diet comprising 75% plants and 25% insects (Peng et al. 2008). Previous studies have documented the ways in which Light‐vented Bulbuls adapt to urban environments through reproductive and physiological mechanisms. For instance, they incorporate anthropogenic materials (e.g., plastic, chemical fiber) into their nests, which enhances the hatching success rate (Chen et al. 2024). Additionally, the gut microbiota composition of Light‐vented Bulbuls changed with latitude, improving their metabolic heat production, which may facilitate their expansion into colder northern cities (Wu et al. 2023). However, behavioral adaptations in the foraging strategies of Light‐vented Bulbuls in urban environments have not yet been documented. In this study, we report two novel foraging behaviors exhibited by the Light‐vented Bulbul. Although the Light‐vented Bulbul is a common bird in urban areas, these behaviors had not been previously observed or documented in any literature. These behaviors exemplify its behavioral plasticity and contribute to an understanding of why it has become a bird well‐adapted to urban environments.

## 2 Observations and Results

**FIGURE 1:**
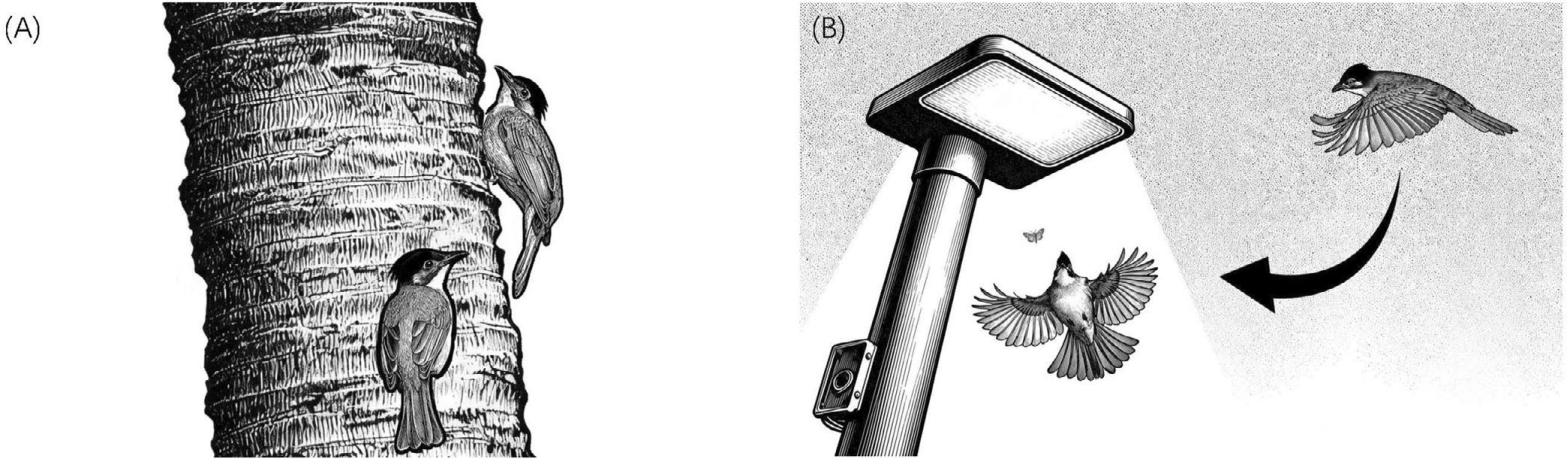
Novel foraging behaviors of Light‐vented Bulbul ( *Pycnonotus sinensis* ). (A) Feeding on insects on the coconut tree trunk like a woodpecker. (B) Preying on moths attracted by the light under the cover of night.

The first behavior observed involves woodpecker‐like foraging (Figure 1A). In March 2025, we observed 5–6 Light‐vented Bulbuls on Hainan Normal University campus in Haikou, Hainan Province, China (19°59′52.55″ N, 110°20′19.19″ E). The Light‐vented Bulbuls were observed vertically climbing coconut palm ( *Cocos nucifera* ) trunks using their claws, bracing themselves with their tail feathers, and pecking, gleaning, and consuming at the trunk surface like woodpeckers (Video S1). Subsequently, we collected insects from the tree trunk and examined them under a stereomicroscope, identifying them as psocids (Order Psocodea, Family Psocidae). The second behavior involves nocturnal foraging under artificial lighting (Figure 1B). On the evening of May 2, 2025, at 19:55 (Beijing local time, UTC + 8), when darkness had fully enveloped the sky, we observed a Light‐vented Bulbul in Linwang Town, Sanya City, Hainan Province (18°18′40.98″ N, 109°41′53.84″ E). The bulbul was perched on a roadside power line and subsequently flew toward a streetlight above, hovering in mid‐air with continuous wing‐flapping as it chased a flying insect (Video S2).

## 3 Discussion

The first foraging behavior we observed suggests that Light‐vented Bulbuls were able to forage insects on tree trunks. With accelerating urbanization and intensified urban management, forests are cleared, and dead trees that serve as potential cavity‐nesting sites are often removed to reduce fire hazards. This has led to a substantial reduction in the availability of suitable nesting sites for cavity‐nesting birds such as woodpeckers (Gottschalk et al. 2011; Monti et al. 2019; Dulle et al. 2020; Miranda et al. 2021). Preliminary bird surveys in Haikou City have also failed to detect the presence of any species of woodpecker (Lin and Liang 2025b), but five species of woodpeckers and one species of nuthatch have been documented in the tropical rainforests of inland Hainan (Lin 2024). Simultaneously, the simplified vegetation structure and monotonous, repetitive artificial tree plantings in cities may provide suitable habitats for certain invertebrate taxa, potentially leading to population outbreaks and pest infestations (Kotze et al. 2022). In Haikou City, for example, coconut palms are extensively used in landscape greening due to their edible fruit and typhoon resistance. In the days following the outbreak of psocids, the fronds of these palms exhibited varying degrees of wilting, suggesting potential disease infestation, with psocids likely contributing to this pathology. Thus, the outbreak of insects may have catalyzed the Light‐vented Bulbul's foraging behavior on tree trunks.

The second foraging behavior demonstrated the Light‐vented Bulbul utilizing streetlights for nocturnal feeding. Although numerous studies have focused on the negative impacts of urban roads and associated infrastructure on wildlife, research on positive effects remains relatively scarce (Fahrig and Rytwinski 2009). For example, artificial light at night (ALAN) from streetlights and vehicle headlights has been shown to negatively affect avian circadian rhythms (Kempenaers et al. 2010; Dominoni et al. 2014), reproduction (Dominoni et al. 2013; Zhang et al. 2019) as well as development, song patterns, and migration (De Molenaar Sanders and Jonkers 2006). However, recent studies have also revealed potential positive effects of roads and lighting on birds (Morelli et al. 2014). For example, Scaly‐breasted Munias ( *Lonchura punctulata* ) frequently construct communal nests on major roadways to deter nest predators (Zhou et al. 2020). Additionally, White Wagtails ( *Motacilla alba* ) and Red‐billed Starlings ( *Spodiopsar sericeus* ) select busy road intersections as nocturnal roosts, utilizing vehicle lights and human activity for predator avoidance (Jiang et al. 2021; Lin and Liang 2025c). In addition to employing artificial light for predator avoidance, light‐assisted predation has also been documented. For example, Short‐eared Owls ( *Asio flammeus* ) and Long‐eared Owls ( *Asio otus* ) prey on migratory songbirds attracted to lights during migration seasons, thereby increasing predation opportunities (Canário et al. 2012). Lesser Kestrels ( *Falco naumanni* ) hunt insects and bats near artificial lights (Negro et al. 2000). In our case, the Light‐vented Bulbul may utilize artificial lighting to improve prey detection conditions. This behavior demonstrates that the Light‐vented Bulbul may have the ability to adapt to urban light pollution; however, more research is needed to accurately quantify its impact on its behavior and fitness.

Although we have documented novel foraging behaviors in Light‐vented Bulbuls, these behaviors currently occur only in a small number of individuals. Unless these behavioral patterns become established through natural selection (Reader et al. 2016) or spread through social transmission and cultural inheritance (Aplin 2016, 2019), they may gradually disappear with the death of these individuals. Nevertheless, our observations provide new insights into the behavioral plasticity of Light‐vented Bulbuls in urban environments. Additional observation and research are necessary to determine whether these novel behaviors have the potential to become established and spread throughout the population, thereby forming a cultural‐like phenomenon.

## Author Contributions

**Yuxin Li:** data curation (equal), formal analysis (equal), investigation (equal), methodology (equal), writing – original draft (equal). **Sidan Lin:** data curation (equal), formal analysis (equal), investigation (equal), methodology (equal), visualization (equal), writing – original draft (equal). **Wei Liang:** conceptualization (equal), funding acquisition (equal), supervision (equal), validation (equal), writing – review and editing (equal).

## Funding

WL was supported by the National Key R & D Program of China (2023YFF1304600).

## Ethics Statement

The experiments comply with the current laws of China. No special permit was required for this study as it was not involved in animal or plant collection.

## Conflicts of Interest

The authors declare no conflicts of interest.

## Supporting information


**Video S1:** Light‐vented Bulbul ( *Pycnonotus sinensis* ) feeds on insects on the coconut tree trunk like a woodpecker.

**Video S2:** Light‐vented Bulbul ( *Pycnonotus sinensis* ) preyed on moths attracted by the light under the cover of night.

## Data Availability

No data used for this study. Observations of novel foraging behaviors exhibited by Light‐vented Bulbuls were provided as Supporting Information.
